# Two cases of nursing older nursing home residents during COVID-19

**DOI:** 10.1177/09697330231185944

**Published:** 2023-08-19

**Authors:** Pier Jaarsma, Petra Gelhaus, My Eklund Saksberg

**Affiliations:** Department of Health, Medicine and Caring Sciences, 4566 Linköping University, Sweden; Department of Psychiatry, 4564 Region Östergötland, Linköping, Sweden; Department of Health, Medicine and Caring Sciences, 4566 Linköping University, Sweden

**Keywords:** Ethics of care/care ethics < theory/philosophical perspectives, COVID-19 isolation, medium-range narratives, nursing homes, codes of ethics <topic areas, empowerment, care of the older person < areas of practice, moral distress < topic areas, ethics and dementia care < topic areas, palliative care < topic areas

## Abstract

**Introduction:**

Two ethical challenges of nursing home nurses during the COVID-19 pandemic in Sweden are discussed in this paper.

**Background:**

Historically, the nurse’s primary concern is for the person who is ill, which is the core of nurses’ moral responsibility and identity. In Sweden, person-centered care is generally deemed important in nursing older nursing home residents.

**Objective:**

To chart moral responsibilities of nursing home nurses in two cases involving older residents during the COVID-19 pandemic in Sweden.

**Methods:**

We used Margaret Urban Walker’s framework for moral responsibilities and the International Council of Nurses (ICN) code of ethics for nurses (2021) for our normative analysis.

**Ethical considerations:**

Written and verbal consent was obtained before the interviews, and information was given that participation was entirely voluntary and possible to cancel at any time before the work was published. The Swedish Ethical Review Agency gave an advisory opinion stating that there were no ethical objections to this research project (Dnr. 2020-05649).

**Findings:**

Case #1: a palliative older nursing home resident who was coercively tested for COVID-19, and case #2: a COVID-19–infected resident with dementia who was isolated using sedation. The decision that was finally made in the respective case was analyzed in the light of either consequentialist/utilitarian or non-consequentialist/deontological reasons.

**Discussion:**

Empowerment of nurses as moral agents is required for the application of practical wisdom in the balancing of different care relationships (responsibilities), moral identities (professional virtues), and competing moral values. This requires resources and opens possibilities for profound ethical reflection in nursing education and at work.

**Conclusion:**

During the COVID-19 pandemic, the moral and professional responsibility of nursing home nurses to deliver person-centered care was sometimes problematically abandoned in favor of a more utilitarian manner of ethical decision-making.

## Introduction

Two of the ethical challenges that were narrated by nursing home nurses in the context of the research project “Nurses” experiences concerning prioritization for health and wellbeing of older nursing home residents during the COVID-19 pandemic in Sweden: a qualitative study’ are discussed in this paper. The first case relates to coerced COVID-19 testing of a palliative patient, and the second case to isolation of a COVID-19–infected person with dementia by means of sedation. In both cases, nursing home nurses’ moral responsibilities, their professional and moral identities, and their possibility to engage in person-centered care are at stake.

Our discussion of these cases is structured as follows. Firstly, in the background section, the ethical challenges for nurses during the COVID-19 pandemic, and the importance of person-centered nursing care are briefly described. Secondly, in the method section, Margaret Urban Walker’s framework of charting moral responsibilities^1^ is introduced. Thirdly, in the findings section, the cases are presented and discussed using Walker’s framework in terms of moral relationships, identities, and values. Fourthly, in the discussion section, a general discussion of the findings and the moral framework is given, followed by a conclusion.

## Background

During the COVID-19 pandemic ethical challenges, such as safety of nurses and others, allocation of resources, and the changing nature of nurses’ relationships with patients and families became apparent.^2^ These and other ethical challenges were also touched upon in the interviews in the earlier mentioned Swedish research project.

Historically, the nurse’s primary concern has been and is for the person who is ill, which is the core of nursing responsibility and moral identity.^3^ Since person-centered care is increasingly emphasized in aged care policies and national guidelines and because of a consensus about its relevance,^4^ person-centered care featured as a major touchpoint for our analysis and discussion.

Insights from these and other cases, especially those pertaining to moral responsibilities, relationships, identities, and values, can inform nurses and nursing practice to help with ethical decision-making for the purpose of good patient care. Moreover, nurse educators could use our approach, using a more particularized lens, to prepare nurses better for difficult patient care situations to prevent moral distress, burnout, and leaving the profession.^5^ Finally, these insights might be mobilized to critique problematic contexts in which nursing care occurs and so may help overcome barriers to good practice.

## Objective

The objective of this paper is to chart moral responsibilities of ethically challenged nursing home nurses in two cases involving older residents during the COVID-19 pandemic in Sweden.

## Methods

We started by selecting two pseudonymized narratives, cases, or events, from the original interview study. The semi-structured interviews (using open-ended questions from an interview guide (to prevent bias)) were conducted by the third author of this paper. Her background is a nurse and PhD-student, and she has also worked as a municipal health care executive. The aim of this study was to explore and describe how nurses set priorities for health and wellbeing of older nursing home residents in Sweden during the COVID-19 pandemic. The interviews (with 21 nursing home nurses) resulted in a dataset of 58 events. Two of these events were particularly salient since the nurses explicitly mentioned that the care in which they were involved “did not feel good,” “it felt like abuse” (case #1), “it does not feel completely ethically right,” and “it doesn’t feel good” (case #2). These expressions elicited us to select the two cases since emotions are “upheavals of thought,” sometimes intelligently pointing to things that matter morally.^6^ Our intuition was that the emotional expressions were linked to sensing a conflict of responsibilities by these nursing home nurses in these cases. In turn, this intuition pointed to the need to explore the moral responsibilities of nursing home nurses in these two cases. The cases were translated and adapted to improve readability and are presented in this paper—not only to illustrate the identified challenges but also to function as medium-range narratives.^7^ “Medium-range” means here that these narratives allow us to focus on both the individual and societal implications of moral decisions without sacrificing one perspective for the other from the very beginning. The cases in this paper are deduced from separate events narrated by two nursing home nurses, respectively, in the study.

We then reflected upon the moral responsibilities of nursing home nurses by using the moral philosopher Margaret Urban Walker’s framework of charting moral responsibilities, focusing on narratives of relationship, identity, and values.^1^ Walker suggests that “we have an urgent need for geographies of responsibility, mapping the structure of standing assumptions that guide the distribution of responsibilities—how they are assigned, negotiated, deflected—in particular forms of moral life.”^1^ A geography of responsibility “is meant to tell us something about responsibilities as assigned and assumed in actual life. […] It tries to map features of our practices of responsibility […] of holding ourselves and others to account and taking ourselves and others to task.”^8^

**Figure 1.**
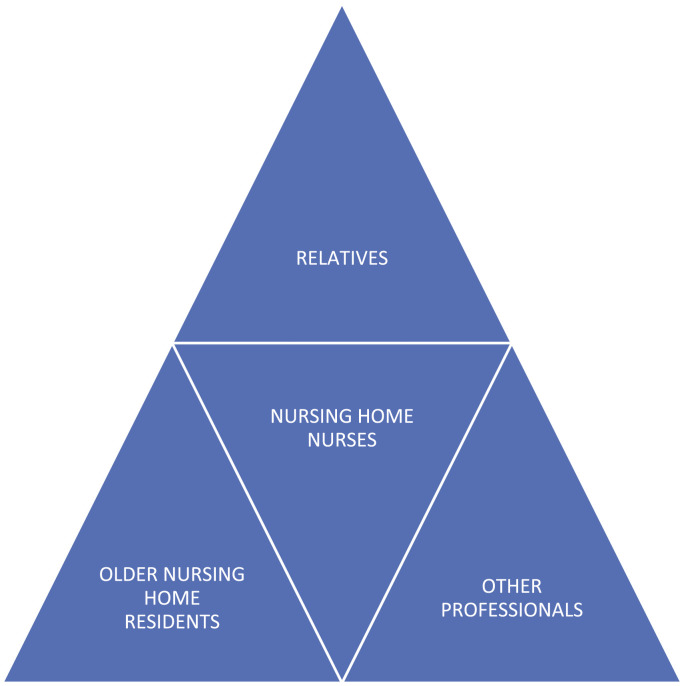
Model of interpersonal care relationships.

We identified the web of interpersonal care relationships surrounding nursing home nurses (Figure 1), in order to sketch a “geography” of moral responsibilities according to Walker’s framework. We chose to situate the narrating nurse in the center of the figure in order to focus on her/his ethical challenges. Ethically, however, the moral dilemma is centered according to professional values surrounding the older person; the one who must bear the main consequences of the decision.

Each case is followed by an ethical reflection starting with Walker’s framework, charting the moral responsibilities of the nursing home nurses in the cases in terms of (1) narratives of care relationships realized by the narrator, (2) narratives of nurses’ professional identity, and (3) narratives of nurses’ professional and moral values. The latter are conceptualized according to the authoritative International Council of Nurses’ Code of Ethics for Nurses.^9^

The decision that was finally made in the respective case is then analyzed in the light of either consequentialist/utilitarian or non-consequentialist/deontological reasons. Such reasons (supporting alternative actions) are presented in this paper, describing the structure and the complexity of the moral responsibilities of nursing home nurses, while highlighting, contrasting, and problematizing their moral decision-making. Legal aspects are not discussed.

## Ethical considerations

Written and verbal consent was obtained before the interviews, and information was given that participation was entirely voluntary and possible to cancel at any time before the work was published. The Swedish Ethical Review Agency gave an advisory opinion stating that there were no ethical objections to the research project (Dnr. 2020–05,649).

## Findings

Two specific cases were identified through immersion, that is, by listening to the recorded interviews and reading/re-reading the transcribed interviews from the research project. We chose these cases because they saliently presented nursing home nurses’ ethical challenges pertaining to the containment of COVID-19 infections (COVID-19 isolation). The first case centers around coercing a palliative Parkinson patient to do a COVID-19 test, while the second is about using sedatives to isolate a COVID-19–positive patient with dementia. The moral decision-making processes included nursing home nurses and other professionals; namely, physicians, and the medically responsible nurse (MAS) of the relevant community. Consecutively, narratives of care relationships, moral identities, and moral values for each case are reflected on, followed by a general discussion.

### Coerced COVID-19 testing

**Case #1: Mr. Johansson and nurse Anna**

We had a Parkinson patient who was very ill and at the end of his life. His breathing was very wheezy. The physician had a palliative breakpoint conversation [i.e., a conversation about moving from active life sustaining treatments to more symptom management therapies] with him, but he was not really conscious. Because of the wheeze the physician thought we might do a Covid test. It did not feel humane for the patient to do the test because this person was not really conscious. He was conscious and yet he wasn’t because he reacted very strongly to the test when I took it. That did not feel good. He tried to defend himself by trying to raise his hands to remove the test swab because he did not really understand what I was doing even if I tried to explain it. It felt like abuse because he did not clearly understand what I was doing and why I was doing it. It was uncomfortable for the patient. I also had to hurry to do the test before the test car drove away. It was necessary to do everything so fast that I did not have time to try to make him understand what I was doing. It wasn’t Covid after all, and the patient died after a couple of days. He would have died anyway because he was at the end of his life, so to speak. At least I could help him get rid of the slime, thankfully. But sometimes you must do certain things that you may not feel comfortable with because the situation requires it. Here, it would not have been good if it had been Covid. You might have exposed relatives to a risk because at that time relatives still were allowed to visit them.

### Care relationships

In Walker’s framework a “narrative of relationship is a story of the relationship’s acquired content and developed expectations, its basis and type of trust, and its possibilities for continuation.”^1^ An important care relationship in this case is the one between the nurse and the patient. The acquired content of the relationship is that the resident—by the very fact that he is a resident in a nursing home—has a legitimate expectation and trust that the nurse will care about him and for him in a professional way. The patient may be unaware of the fact that the nurse’s care relationship with him is not the only care relationship that matters to the nurse in that moment. Perhaps also because of a perceived pressure to make haste, due to a soon to be leaving test-car, the nurse is unable to explain to him that the COVID-19 test is necessary to prevent relatives, other residents, and nursing home personnel from becoming ill. Accordingly, other care relationships will have weighed in on the choices and actions of the nurse—which was implicitly mentioned in the nurse’s narrative when she stated: “because the situation requires it.” One of the care relationships that the nurse may have considered is the one to the rest of society. She may feel she has a responsibility to society at large to stop the spread of COVID-19. So basically, the nurse has the choice to compromise her care relationship to the patient by carrying on with the test, even though she understands that the patient does not want the test, or to compromise other care relationships; namely, the ones to the resident’s relatives, to other residents, to other nursing home personnel, and to society at large, by not executing the test. Her de facto choice was to strain the care relationship to the patient. She regrets letting him down: “It felt like abuse.” In her reflection on this event, she nevertheless defends this “abuse” and deflects her responsibility for this patient by stating that “Sometimes you have to do certain things that you may not feel comfortable with because the situation requires it” and by stating hypothetically that if the patient did have COVID-19, their relatives would have been exposed to a risk of contracting that disease. Strikingly, the nurse only refers to the relatives of this resident without mentioning others that might have run the risk of being contaminated by the patient.

### Identity

Walker understands a narrative of moral identity as “a persistent history of valuation that can be seen in a good deal of what a person cares for, responds to, and takes care of.”^1^ The nurse’s identity risks being changed from being a provider of person-centered care, which is demanded in article 1.10 of the ICN code of ethics for nurses,^9^ to someone who places the health and wellbeing of the many over that of the individual. From a dualistic ethical perspective with respect to actions, the nurse can either act in a consequentialist or in a non-consequentialist manner. A typical ethical consideration includes both consequentialist and non-consequentialist (deontological/Kantian), and maybe many other ethical–theoretical perspectives. Simplified discussions often focus on the dichotomy between consequences and moral duties. In our discussion, we use the dichotomy to highlight the ethical challenge, but at the same time, we use an ethical framework to broaden the view in a systematic way. In the above case, the nurse chooses not a non-consequentialist (deontological/Kantian) stance but a consequentialist one. Very roughly speaking, in non-consequentialism, it is forbidden to harm an individual if it is just for the good of the many and not also for the benefit of that individual. In consequentialism, however, it is allowed and even morally obligatory to harm an individual if the overall utility is maximized by doing so. In this case, if the nurse would have engaged in Kantian (non-consequentialist) reasoning, she could have invoked the first formulation of the categorical imperative: “Act only in accordance with that maxim through which you can at the same time will that it become a universal law.”^10^ Harming an individual solely for the good of other individuals is clearly not compatible with this first formulation of the categorical imperative. According to Immanuel Kant, it would be irrational to make the maxim “harm an individual if it maximizes the good of all other individuals” a universal law. The second formulation of Kant’s categorical imperative; “Act in such a way that you treat humanity, whether in your own person or in the person of any other, never merely as a means, but always at the same time as an end,”^11^ is perhaps even more clear in this case, since testing a very ill patient who is at the end of his life, and who, moreover, indicates by vehemently trying to remove the test swab that he does not wish to be tested, means treating the humanity in this person merely as a means to the end of preventing further contamination, without treating him at the same time as an end; that is, with the patient’s best at heart. “It did not feel good” and “it felt like abuse” relates to this deontological ethical demand, in which not respecting the humanity of an individual patient is categorically rejected. Also, a human rights approach could support this line of reasoning. For instance, article 5 of the United Nations’ Universal Declaration of Human Rights could be invoked in that it, among other things, forbids inhuman treatment.^12^

However, a consequentialist ethical viewpoint appears to have been given the greatest weight in the nurse’s reasoning. Intuitively, the nurse’s choice did not feel good to her, but she attempts to justify her actions by referring to the specific requirements of the situation; particularly preventing the patient’s relatives from being exposed to the COVID-19 virus. By formulating this consequentialist justification, the nurse tries to maintain her moral and professional identity and integrity as a nurse. However, she runs the risk of losing her identity as a nurse who provides person-centered care. She must change her identity to one who is willing to sacrifice person-centered care for the benefit of others whenever “the situation requires it,” for example, when society, the organization, or a physician demands it. The moral choice the nurse has made and the ensuing possible change in moral identity may be a source of moral distress possibly resulting in mental health problems, burn-out, and leaving the profession prematurely.

### Values

The narrative of moral values spans and supports the narratives of relationship and identity, which inevitably intertwine. It is “a history of our shared understandings of what kinds of things, relationships, and commitments really are important, and what their relative importance is.”^1^ Moral and professional nursing values are summed up in the International Council of Nurses code of ethics for nurses: “respect, privacy, advocacy, competence, service, leadership, expertise, integrity, judgement, knowledge, public good, accountability, responsibility, collaboration, responsiveness, compassion, dignity, care, inclusivity, empathy, fairness, justice, equity, solidarity, trust, skill, confidentiality, and safety.”^9^ Next to the rather obvious value of good judgment, the prima facie-relevant values in this first case are respect, public good, responsibility, collaboration, responsiveness, compassion, dignity, care, empathy, solidarity, trust, and safety. The nurse has given a different weight to each of these values in the action alternatives available for the nurse, that is, whether to coercively execute the COVID-19 test.

The first action alternative of refraining from coercively executing the test is supported by the professional values of respect, responsiveness, compassion, dignity, care, empathy, and trust. The professional value of respect refers to respecting the autonomy of the patient. One could argue that the patient (by raising his hands to remove the test swab) shows his will that he does not wish to be tested and that his autonomy therefore should be respected. Against this, one could argue that this patient is not fully autonomous anymore. His autonomy is compromised by the fact that he is semi-conscious. It may further be argued that the patient is only expressing a sub-conscious reflex in trying to prevent the COVID-19 test swab from entering his nostril. Or one could argue that even if he were conscious and if he had received sufficient information about the necessity of the test, he probably would have consented to it.

Responsiveness to the needs of this individual patient, compassion with this patient’s suffering, and respect for his dignity at the end of his life, and the patient’s trust in those that care for him, are all values that play a role in this first action alternative.

The second action alternative to coercively executing the test, which was chosen by the nurse, is primarily supported by the values of public good, responsibility, collaboration, solidarity, trust, and safety, creating a narrative of value that may justify the nurse’s choice. Testing for COVID-19 was primarily justified by the desire to prevent further spread of a potentially lethal disease. This justification was formulated by the nurse when she referred to the risk relatives run when they come to visit this resident. Both action alternatives are supported by the values of responsibility and safety, but these values are aimed at different subjects and weighed differently. In the first action alternative, responsibility and safety are primarily aimed at the patient, that is, the nurse is responsible for the safety of this individual patient. In the second action alternative, these values are aimed at the public good, that is, the nurse is responsible for the safety of the wider public. Moreover, the nurse’s choice shows that in this situation, these values are given a greater weight when aimed at the wider public. Trust as a professional value also plays a role in the justification of this second action alternative, because the wider public also needs to be able to trust a nurse to take their interests into account by preventing COVID-19 from further spread. Finally, collaboration can be identified as a relevant value in the nurse’s justification of the second action alternative, because the nurse felt she needed to comply with the physician’s order to execute the test, at which she hinted when she stated “[b]ecause of the wheeze the physician thought we might do a COVID-test.” The nurse’s responsibility to collaborate with the physician and even comply with the physician’s prescription is assigned to her by the tradition of the nurse–physician relationship and appear to be non-negotiable.

## Isolation using sedation

**Case #2: Mrs. Karlsson and nurse Johanna**

Mrs. Karlsson was our first COVID-19 patient. She had dementia and wandering behavior. It was difficult to keep her in her room. The medically responsible nurse (MAS) of the community and the patient-responsible physician (PAL) became involved and it was decided that we should simply sedate her so that she would not go out and be a risk of infection to other patients. There it would have been better if we had a COVID-19 ward, alternatively put in some extra staff who are in with her all the time and can slow her down in time. In retrospect, it does not feel completely ethically right, but you do what your superiors say, both doctors and MAS. It doesn’t feel good to sedate a person who is not aware of herself just so she wouldn’t go out and infect others. In this particular situation, it felt like it was us, the nurses’ responsibility, and that sedation was the only option, but it feels like if the unit manager would have gotten in more staffing—maybe put in some wake or some extra staff who have social interaction with her and are there all the time, just to be able to limit her wandering—we could have avoided sedating the patient. It would have felt better then, ethically. In the end, I think it was the best thing for the patient, but somehow it didn’t feel right. As a nurse, we should think a lot about the well-being of patients and their right to things, but it felt a bit like they skipped it, that there was too much focus on the spread of infection, unfortunately.

### Care relationships

In this case, care relationships between the nurse and the resident, between the nurse and other residents, between MAS and PAL and the resident, between MAS and PAL and the other residents, and between the manager and the resident are referred to in the nurse’s narrative. Just as in the first case, the acquired content of the relationship between the nurse and the resident is that the resident—by the very fact that she is a resident in a nursing home—has a legitimate expectation and trust that the nurse will care about her and for her in a professional way. The resident may be unaware of the fact that the nurse’s care relationship to her is not the only care relationship that matters to the nurse. The nurse is unable to prevent the person with dementia from wandering around the house. Her care relationship to other residents is at risk of becoming compromised, and therefore she involves MAS and PAL. They also have care relationships with the other residents, and they direct the nurse to sedate the resident so that she does not wander any longer and the risk that other residents will be infected with COVID-19 is minimized. The nurse may also have considered, but does not mention, the care relationships to the relatives of the residents, the staff caring for the residents, and the rest of society. She may feel she has a responsibility to society at large to stop the spread of COVID-19. So basically, the nurse in this case has the choice of either compromising her care relationship with the patient by sedating her—even though she understands that the patient is unaware of the reason for this sedation and will likely not benefit from it herself—or to compromise care relationships with others who run the risk of being infected by COVID-19 if this resident cannot be convinced to stay in her room voluntarily. The choice the MAS and PAL made, and which was initially accepted by the nurse, runs the risk of straining her care relationship with the patient and she feels bad about it: “[i]t doesn’t feel good to sedate a person who is not aware of herself.” In her reflection on this event, she tries to deflect her moral responsibility for sedating this patient by stating “you do what your superiors say.” Moreover, she defends the choice that was made by the MAS and PAL by stating “[i]n the end, I think it was the best thing for the patient,” probably referring to the alternative of restraining the resident by other physical means, and not referring to the possibility that this patient could directly benefit from sedation. Contradicting the nurse’s assessment of sedation as being in the patient’s best interest is her assertion that extra staff would have been better for this patient. In stating this, she deflects her responsibility to that of the unit manager to supply sufficient personnel.

#### Identity

The nurse tries to maintain her identity as somebody who provides person-centered care by stating “as a nurse, we should think a lot about the well-being of patients and their right to things, but it felt a bit like they skipped it, that there was too much focus on the spread of infection, unfortunately.” Again, applying the second formulation of Kant’s categorical imperative, it becomes clear that Mrs. Karlsson is merely treated as a means to an end, that is, sedating her to prevent further COVID-19 infections, and not at the same time *as* an end, that is, as an individual human being. Treating her as an end, which is at the core of person-centered care, would demand that she somehow benefited by sedating her. However, the nurse’s narrative does not reveal how Mrs. Karlsson benefited by being sedated, if ever she was. With respect to person-centered care, the nurse clearly deflects her responsibility for not following Kant’s categorical imperative and consequently for failing to provide person-centered care by stating “they [i.e., MAS and PAL] skipped it [i.e., person-centered care].” Accordingly, in this case, the nurse’s identity also risks being changed from being a provider of person-centered care to someone who puts the health and wellbeing of the many over that of an individual, as indicated by the statement “we should simply sedate her so that she would not go out and be a risk of infection for other patients.” But unlike nurse Anna, who appears to accept the departure from person-centered care, nurse Johanna vehemently rejects it stating that wellbeing and the rights of Mrs. Karlsson were ignored and that “there was too much focus on the spread of infection.” It seems that nurse Johanna has a stronger person-centered care identity than nurse Anna. Nurses with a stronger person-centered care identity may have a higher risk of experiencing moral distress than nurses with a lower one when they feel they must compromise person-centered care. In turn, a stronger person-centered care identity may also result in a higher risk of burn-out and of leaving the profession prematurely. This may be enforced if the nurse has acquired knowledge of the fact that person-centered care has been shown to have a beneficial effect on agitation of people with dementia.^13^ On the other hand, Johanna may have had a stronger orientation and capacity to discuss moral decision-making. Given time and opportunity for this, she might become a stronger moral agent, who develops the workplace and has the potential for a stronger and more stable identification with decision-making, which implies less moral distress.^14^ Anyhow, in case of moral challenges, moral distress,^15^ and threats to moral identities, the question is raised as to whether the person-centered care paradigm should be adjusted for times of crisis like that of the COVID-19 pandemic, or whether nurses need to be prepared for such an adjustment; for even though person-centered care is the paradigm, it may not always be possible to follow that paradigm.

#### Values

The first action alternative of refraining from sedating a patient with dementia in order to stop her wandering behavior—that is,, to isolate her to prevent the spread of COVID-19 infections—is supported by the professional values of respect, responsiveness, compassion, dignity, care, empathy, trust, and safety. Respecting the patient’s freedom of movement can ultimately be based on the principle of respect for autonomy,^16^ although the patient’s autonomy is seriously impaired because of her dementia. Responsiveness to the needs of this individual patient, her need for balance between activity and rest, becomes problematic when sedation is used solely to stop this patient’s wandering behavior. Even in this case, compassion and empathy with this patient’s suffering, respect for her dignity at the end of her life, and this patient’s trust that those that care for her have her best interests at heart, are all values that play a role in this first action alternative to refrain from sedating the patient. Patient safety supports this action alternative, because sedatives are known to increase the risk of falls and fractures in the elderly.^17^

The second action alternative to sedating a patient with dementia in order to stop her wandering behavior and, in turn, to prevent the spread of COVID-19 infections (the action chosen by the nurse) is supported by the professional values of public good, accountability, responsibility, collaboration, fairness, justice, trust, and safety, creating a narrative of value that may justify the nurse’s choice. Isolation in case of a COVID-19 positive patient is only justified by the desire to prevent further spread of a potentially lethal disease. This justification was formulated by the nurse when she stated, “it was decided that we should simply sedate her so that she would not go out and be a risk of infection for other patients.” Unlike in the first case, both action alternatives are not supported by the values of responsibility and safety. These values are not aimed at the patient—on the contrary, these values are compromised because sedatives increase the risk of falls and therefore lower a patient’s level of safety. The nurse is responsible for the safety of this individual patient, but by sedating her she appears to have let this patient down with respect to her safety. In this second action alternative, the values of responsibility and safety are solely aimed at the public good, that is, the nurse as responsible for the safety of the wider public (including other residents). Just as in the first case, trust as a professional value also plays a role in the justification of this second action alternative, because the wider public also needs to be able to trust a nurse to take their interests into account by preventing COVID-19 from spreading further into society. Finally, collaboration can be identified as a relevant value in the nurse’s justification of the second action alternative, because the nurse felt she needed to comply with the MAS’ and PAL’s order to sedate this patient, which she hinted at when she stated, “you do what your superiors say, both doctors and MAS,” referring to the non-negotiable character of her cooperation with both the MAS and the PAL. She later appears to regret this choice, however, when she states, “we could have avoided sedating the patient” and deflects her responsibility to the unit manager who, in the nurse’s view, could have called in more staffing, which might have avoided this situation. Whether this deflection of moral responsibility is justified is unclear, since the provision of more staff may have been problematic, especially during the COVID-19 pandemic, as elderly care in Sweden is “an under-resourced part of society that is staffed by an undervalued team of professionals.”^18^

## Discussion

The above narratives of care relationships, identity, and values sketch a “geography” of responsibilities^1,8^—in this instance, responsibilities pertaining to moral life, when nurses had to decide whether to coerce a resident with end-stage Parkinson’s disease, into taking the COVID-19 test, or whether to follow an order to sedate another resident with dementia in order to stop her wandering behavior and prevent further spread of COVID-19. Important landmarks in this “geography” are the nurses’ responsibilities to cooperate with physicians, to deliver person-centered care, to maintain patient safety, and to help maintain public safety. Trade-offs were made between patient and public safety, to the detriment of the former. It may seem easier to analyze the ethical trade-off merely in terms of deontology and consequentialism, or in the conflict of the moral principles of autonomy and justice. It could be attractive to concentrate on the person-centered care responsibility of the nurse and leave the public health responsibility to other agents. This solution, however, comes at a high price. The nurse may feel obliged to act as an executor of orders with which she herself does not agree. The relative powerlessness of nurses in their relationships with physicians (including PALs), MAS, and unit managers is salient in these cases. Nursing home nurses’ professional and moral responsibilities appear to be rigidly assigned, non-negotiable, and sometimes nurses feel the need to deflect their sense of responsibility to another professional—for example, the unit-manager—or more abstractly to the conditions of a particular situation, which at first sight appears to be a coping mechanism intended to lower their moral distress. In either case, moral distress is a necessary consequence of this strategy. The decision made is removed from the resident, and the nurse is the one who must act according to the decision in direct conflict with the patient. It is the nurse who feels the dark side of the ethical dilemma in her own body and must deal with the consequences if her restrictively person-centered care is compromised. In an Aristotelian light, she is forced to compromise her own identity as a virtuous person or at least as a good nurse.^19^

Mapping the ethical reflection in terms of care relationships, professional identity, and professional values has, however, the potential to go deeper into the dilemmas in a way that is not merely dichotomous. Even if the conflict of interest between the individual resident and the collective interests are at stake, the description of the different relationships involved (1), of the impact on the moral identity of the nurse (2), and of the complicated way the different professional values are addressed (3), contributes to a more complex and differentiated understanding of the problem. Although guides for ethical decision-making in nursing care generally focus on the individual patients and their good over likely harms to them and others involved and may fruitfully be applied to some ethically problematic nursing situations, in other situations nurses’ moral identity and care relationships are in danger of being compromised. These ethical decision-making models do not systematically address consequences for nurses’ moral identities and care relationships.^20^ In contrast, our approach of reflecting on medium-range narratives does systematically address such consequences. Therefore, using this more particularized lens may lead to a deeper and better understanding of action alternatives in ethical decision-making and of the consequences of these alternatives. Ultimately, such better understanding will promote good patient care.

The nurses’ actions imply a need for ethical empowerment, which may be facilitated by (nurse) managers, since one of the managers’ responsibilities is to support nurses in solving ethical problems.^21^ Ethical empowerment is needed both for the nurses’ own sake and for the sake of patient safety and safeguarding person-centered care. At the same time, the nurse cannot be regarded as the (only) advocate for the patient in the situation. Even if the individual care relationship is a powerful moral argument, the nurse herself must weigh it against her other relational responsibilities, which includes possible consequences for other residents, relatives, personnel, and society. The realization that the moral agent must consider her own professional identity is a strong argument against deflecting the inconveniences of decision-making to other agents. The professional values, as mirrored by the ICN, shed light on responsibilities that aim to not only exclusively benefit the individual patient, even if this is the moral core of nursing care.

## Conclusion

The resulting “geographies” of responsibilities, as presented in this paper, although empirically informed, are only sketches. Nevertheless, we believe these sketches can provide valuable insights into the form of moral life and moral dilemmas of nurses caring for older nursing home residents. Insights into how moral responsibilities are assigned, negotiated, and deflected in ethically problematic situations as encountered by nursing home nurses during the COVID-19 pandemic in Sweden. We concluded that during the COVID-19 pandemic the moral and professional responsibility of nursing home nurses to deliver person-centered care was sometimes problematically abandoned in favor of a more utilitarian manner of ethical decision-making. Our application of Margaret Urban Walker’s framework to these situations fosters moral agency of nurses by deepening their knowledge of how moral relationships, identity, and values could be at stake in such situations.

Our analysis also led us to conclude that the nurses in these cases, and similar cases, need to be empowered as moral agents. This, however, requires time and possibilities for structural ethical reflection both in education and continuously at work. It is important that the implementation of structured ethical reflection is a common project in the nursing home, including, for example, PAL, MAS, and representatives of the organization, in order to make ethical decision-making a well-founded and shared process, and not a competition between different stakeholders.

Another observation is that the moral and professional values, as stated in the ICN code of ethics for nurses, could be invoked for either of the opposing action alternatives in the moral dilemmas faced by these nurses. Every moral agent involved needs practical wisdom to apply and weigh these moral values and choose an action alternative. The ICN code of ethics for nurses does not explicitly address the application of nursing’s professional and moral values in times of crisis and limited resources, although a need for this exists, as was made clear in the moral dilemmas in this paper.
